# Supplementary material for: Growth and Weight Status in Chinese Children and Their Association with Family Environments
Source: Children (Basel). 2021 May 14;8(5):397. doi: 10.3390/children8050397 (PMC8157162; doi:10.3390/children8050397)
Supplement: Supplementary file 1 [file children-08-00397-s001.zip › children-1178472-supplementary.pdf]

children

MDPI

Table S1. Comparison of Chinese growth guidelines and the WHO growth reference.

	Height				Weight				BMI				Underweight				Overweight				Obesity			
	Boy		Girl		Boy		Girl		Boy		Girl		Boy		Girl		Boy		Girl		Boy		Girl	
Age	WHO	China	WHO	China	WHO	China	WHO	China	WHO	China	WHO	China	WHO	China	WHO	China	WHO	China	WHO	China	WHO	China	WHO	China
0	67.6	68.4	65.7	66.8	7.9	8.4	7.3	7.8	17.3		16.9		14.7		14.1		20.5		20.3		22.3		22.3	
1	82.3	82.7	80.7	81.5	10.9	11.3	10.2	10.7	16.1		15.7		13.9		13.3		19.0		18.8		20.8		20.8	
2	91.9	93.3	90.7	92.1	13.3	13.6	12.7	13.1	15.8		15.5		13.6		13.2		18.6		18.5		20.2		20.4	
3	99.9	100.6	99.0	99.4	15.3	15.6	15.0	15.2	15.4		15.3		13.2		12.9		18.2		18.4		19.8		20.4	
4	106.7	107.7	106.2	106.7	17.3	17.8	17.2	17.2	15.3		15.3		13.0		12.7		18.2		18.7		20.0		20.8	
5	112.9	114.7	112.2	113.5	19.4	20.2	19.1	19.3	15.3		15.2		14.1		13.9		16.7		16.9		18.4		19.0	
6	118.9	120.7	118.0	119.4	21.7	22.5	21.2	21.4	15.4	15.5	15.3	15.0	14.1	13.8	13.9	13.3	16.9	16.7	17.1	16.5	18.7	18.1	19.5	18.0
7	124.5	125.5	123.7	124.1	24.1	25.7	23.6	23.9	15.6	15.8	15.5	15.1	14.3	13.9	14.0	13.5	17.2	17.4	17.5	17.2	19.3	19.2	20.1	19.0
8	129.9	130.7	129.5	129.3	26.7	28.9	26.6	26.7	15.9	16.2	15.9	15.4	14.5	14.0	14.3	13.7	17.7	18.1	18.0	18.1	20.1	20.3	21.0	19.9
9	135.2	135.8	135.5	134.9	29.6	32.1	30.0	29.9	16.2	16.7	16.3	15.8	14.8	14.2	14.6	13.9	18.2	18.9	18.7	19.0	20.9	21.4	22.0	21.0
10	140.4	140.8	141.8	141.2	32.9	35.6	34.0	33.8	16.7	17.2	16.9	16.4	15.1	14.6	15.1	14.1	18.8	19.6	19.4	20.0	21.9	22.5	23.1	22.1
11	146.0	146.0	148.2	147.4	36.7	40.0	38.7	38.4	17.2	17.8	17.6	17.1	15.5	15.1	15.6	14.5	19.5	20.3	20.3	21.1	23.0	23.6	24.3	23.3
12	152.4	152.2	154.0	152.4	41.5	45.1	43.6	42.9	17.9	18.4	18.4	17.8	16.1	15.6	16.3	14.9	20.4	21.0	21.3	21.9	24.2	24.7	25.6	24.5
13	159.7	160.2	158.3	156.1	47.5	50.9	48.1	46.4	18.6	18.9	19.2	18.5	16.7	16.1	16.9	15.6	21.3	21.9	22.3	22.6	25.3	25.7	26.8	25.6
14	166.3	165.6	160.9	157.8	53.6	55.4	51.5	49.0	19.4	19.4	19.9	19.1	17.3	16.7	17.5	16.3	22.2	22.6	23.1	23.0	26.5	26.4	27.8	26.3
15	171.1	169.0	162.2	158.5	59.0	58.4	53.9	50.5	20.1	19.9	20.5	19.5	18.0	17.0	18.0	16.8	23.1	23.1	23.8	23.4	27.4	26.9	28.6	26.9
16	174.2	170.6	162.7	158.9	63.2	60.1	55.3	51.1	20.8	20.3	20.9	19.9	18.5	17.5	18.3	17.1	23.9	23.5	24.3	23.7	28.3	27.4	29.1	27.4
17	175.8	171.4	163.0	159.2	66.2	61.1	56.2	51.3	21.4	20.7	21.2	20.2	19.0	17.9	18.5	17.3	24.6	23.8	24.6	23.9	29.0	27.8	29.4	27.8
18	176.4	171.4	163.1	160.0	68.3	61.4	56.8	51.4	22.0	20.8	21.3	20.3	19.4	17.9	18.6	17.3	25.2	24.0	24.9	24.0	29.5	28.0	29.6	28.0

Note: Chinese Weight and BMI references are based on 2009 standard [23], while others are updated using the 2018 standard published by the NHC (National Health Commission of the People's Republic of China [24–26]). WHO growth reference are transformed from month-specific growth reference [20,21], where 0 year old refer to the 6 month reference, and 1 year old refers to the 18-month reference.

Table S2. Association between family environments and children' growth status.

	OLS			RE			Logit		
	**height_sd**	**weight_sd**	**bmi_sd**	**height_sd**	**weight_sd**	bmi_sd	underweight	overweight	obesity
age	0.0013***	-0.0054***	-0.0086***	0.0017***	-0.0048***	-0.0086***	0.1011***	-0.0613***	-0.1623***
	0.000	0.000	0.000	0.000	0.000	0.000	(0.004)	(0.007)	(0.009)
female	0.0019**	-0.0101***	-0.0139***	0.0020*	-0.0076**	-0.0116***	0.0376	-0.2219***	-0.5475***
	(0.001)	(0.003)	(0.002)	(0.001)	(0.003)	(0.003)	(0.039)	(0.065)	(0.100)
han	0.0138***	0.0294***	0.0047	0.0142***	0.0300***	0.0041	-0.0246	0.2572**	0.2750*
	(0.001)	(0.003)	(0.003)	(0.001)	(0.005)	(0.004)	(0.054)	(0.103)	(0.155)
Fat_share	0.0284***	0.0489***	-0.0046	0.0239***	0.0519***	0.0049	0.3889**	0.0052	0.3224
	(0.004)	(0.013)	(0.012)	(0.004)	(0.012)	(0.011)	(0.189)	(0.318)	(0.438)
Pro-tein_share	0.1545***	0.5271***	0.2533***	0.1299***	0.4163***	0.1915***	-1.8622**	3.3513***	5.4870***
	(0.017)	(0.058)	(0.050)	(0.015)	(0.048)	(0.045)	(0.833)	(1.175)	(1.641)
urban	0.0003	0.0076**	0.0065**	0.0026**	0.0123***	0.0068**	-0.2460***	0.0690	0.0396
	(0.001)	(0.003)	(0.003)	(0.001)	(0.004)	(0.003)	(0.048)	(0.075)	(0.110)
children	-0.0074***	-0.0200***	-0.0076***	-0.0055***	-0.0178***	-0.0086***	0.1433***	-0.1121**	0.0589
	(0.001)	(0.002)	(0.002)	(0.001)	(0.002)	(0.002)	(0.029)	(0.056)	(0.083)
hhsize	-0.0011***	-0.0029***	-0.0005	-0.0014***	-0.0030**	0.0000	-0.0326*	-0.0882***	-0.0182
	0.000	(0.001)	(0.001)	0.000	(0.001)	(0.001)	(0.017)	(0.028)	(0.038)
ln(income)	0.0029***	0.0073***	0.0017	0.0024***	0.0063***	0.0019*	0.0079	0.0518	0.0043
	0.000	(0.001)	(0.001)	0.000	(0.001)	(0.001)	(0.018)	(0.034)	(0.050)
tapwater	0.0004	-0.0027	-0.0044*	0.0031***	0.0013	-0.0047*	0.1096**	-0.1041	-0.1316
	(0.001)	(0.003)	(0.003)	(0.001)	(0.003)	(0.003)	(0.045)	(0.078)	(0.114)
toilet	0.0038***	0.0080**	0.0005	0.0039***	0.0116***	0.0033	0.1204**	0.0790	0.2384**
	(0.001)	(0.003)	(0.003)	(0.001)	(0.003)	(0.003)	(0.051)	(0.079)	(0.112)
no excreta	0.0092***	0.0229***	0.0067***	0.0054***	0.0105***	0.0010	-0.1288***	0.0437	0.2751**
	(0.001)	(0.003)	(0.002)	(0.001)	(0.003)	(0.002)	(0.044)	(0.074)	(0.110)
Max_edu_parent	0.0022***	0.0044***	0.0004	0.0020***	0.0044***	0.0004	0.0095	0.0367***	0.0155
	0.000	0.000	0.000	0.000	0.000	0.000	(0.006)	(0.012)	(0.017)
Max_BMI_parent	0.0029***	0.0186***	0.0143***	0.0023***	0.0153***	0.0124***	-0.1993***	0.1543***	0.2135***
	0.000	(0.001)	(0.001)	0.000	(0.001)	0.000	(0.009)	(0.011)	(0.017)
Constant	0.8026***	0.3688***	0.7197***	0.8193***	0.4595***	0.7677***	2.2096***	-6.2241***	-8.0974***
	(0.005)	(0.018)	(0.016)	(0.005)	(0.017)	(0.015)	(0.257)	(0.402)	(0.599)
Obs	15259	15259	15259	15259	15259	15259	15259	15259	15259
F/chi2	262.48***	219.64***	153.72***	2783.24***	2385.17***	2104.03***	1116.77***	472.30***	626.98***

Note: p value in brackets. *, ** and *** refer to $p < 0.1$, $p < 0.05$ and $p < 0.01$ respectively. fat_share and protein_share refer to share of energy drawn from fat and protein, children refers to number of children, hhsize refers to household size, ln(income) is measured by household income per capita in logarithm, tapwater indicates in-house or in-yard tap water, toilet indicates in-house flush or in-house toilet, no excreta refers to no near house excreta removal; Max_edu_parent and Max_BMI_parent indicate the maximum education and BMI of parents.

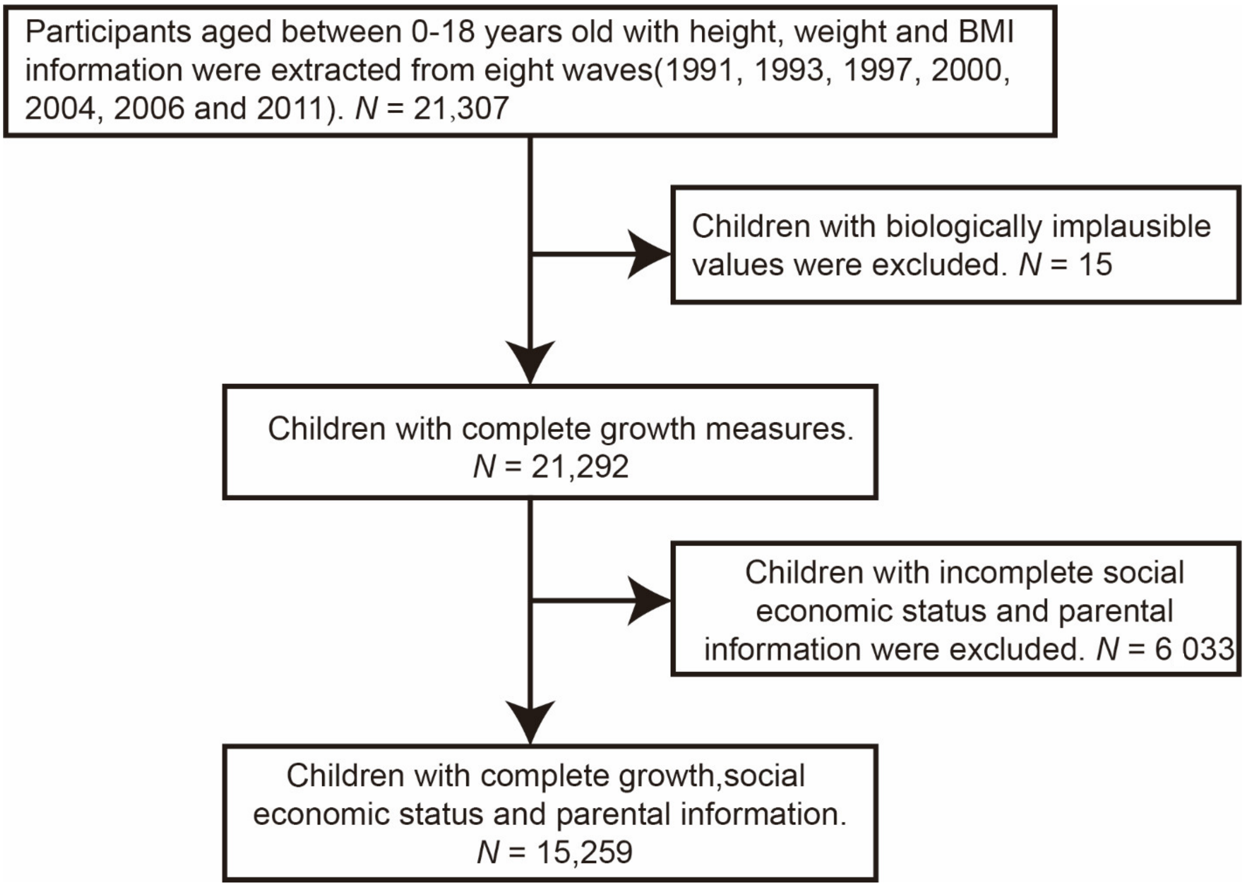

Figure S1. Flow chart.

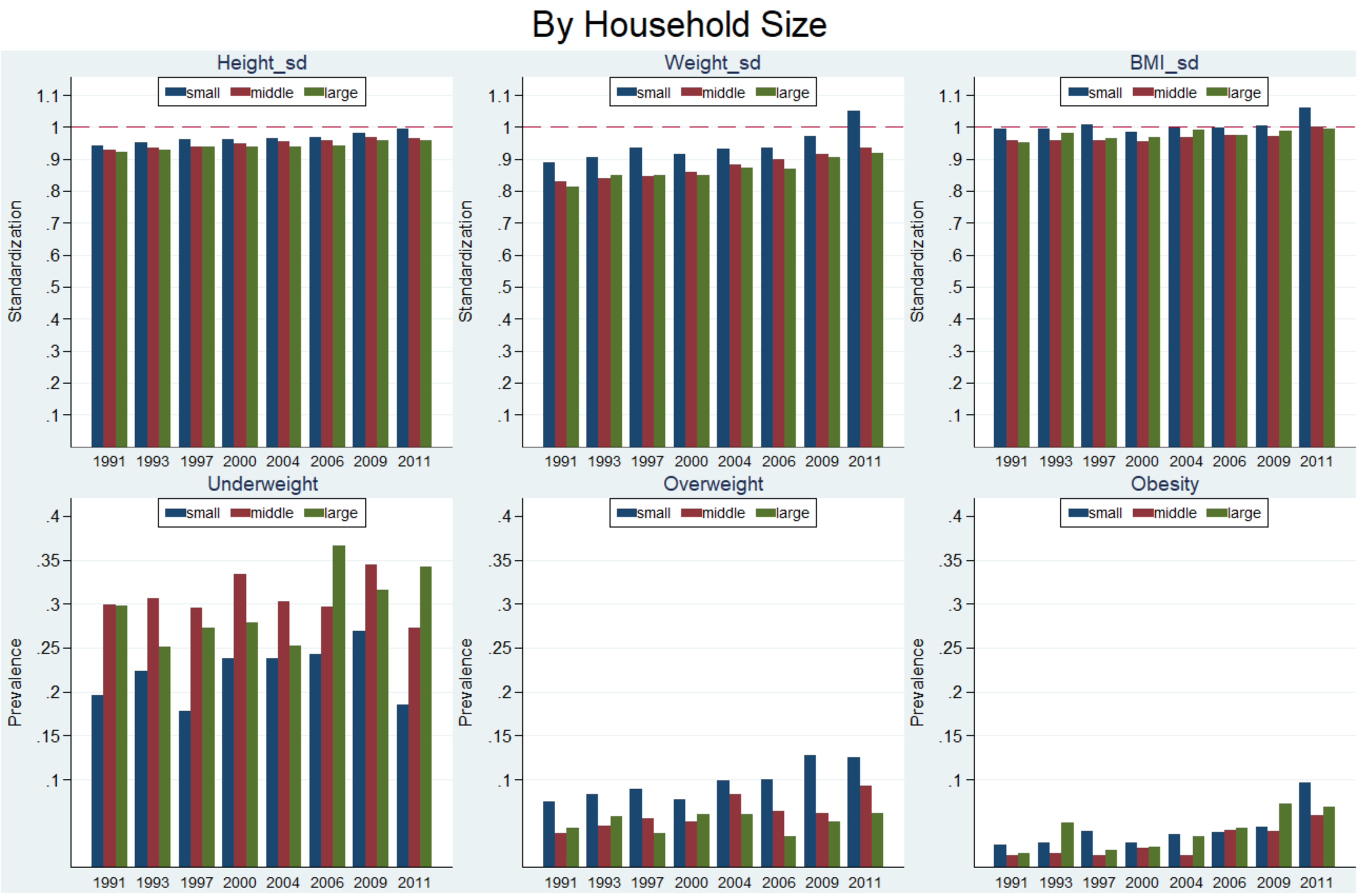

Note: Small, middle and large refer to household with only one child, more than one children but no more than 5 members, and more than one children and more than 5 members.

Figure S2. Secular trend of children's growth indicator and weight status by household size.

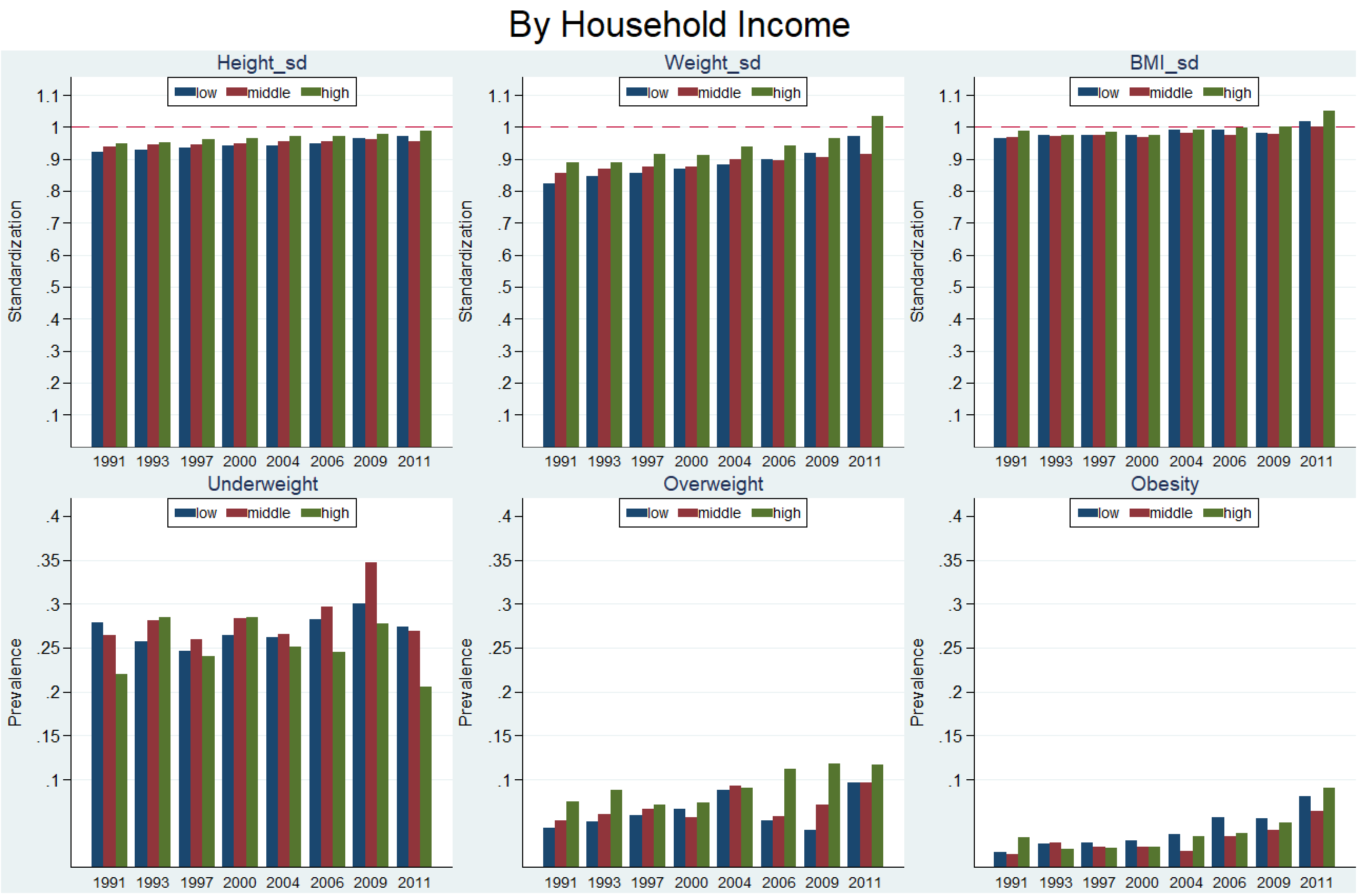

Note: Low, middle, and high refer to three equal income fractals.

Figure S3. Secular trend of children's growth indicator and weight status by household income.

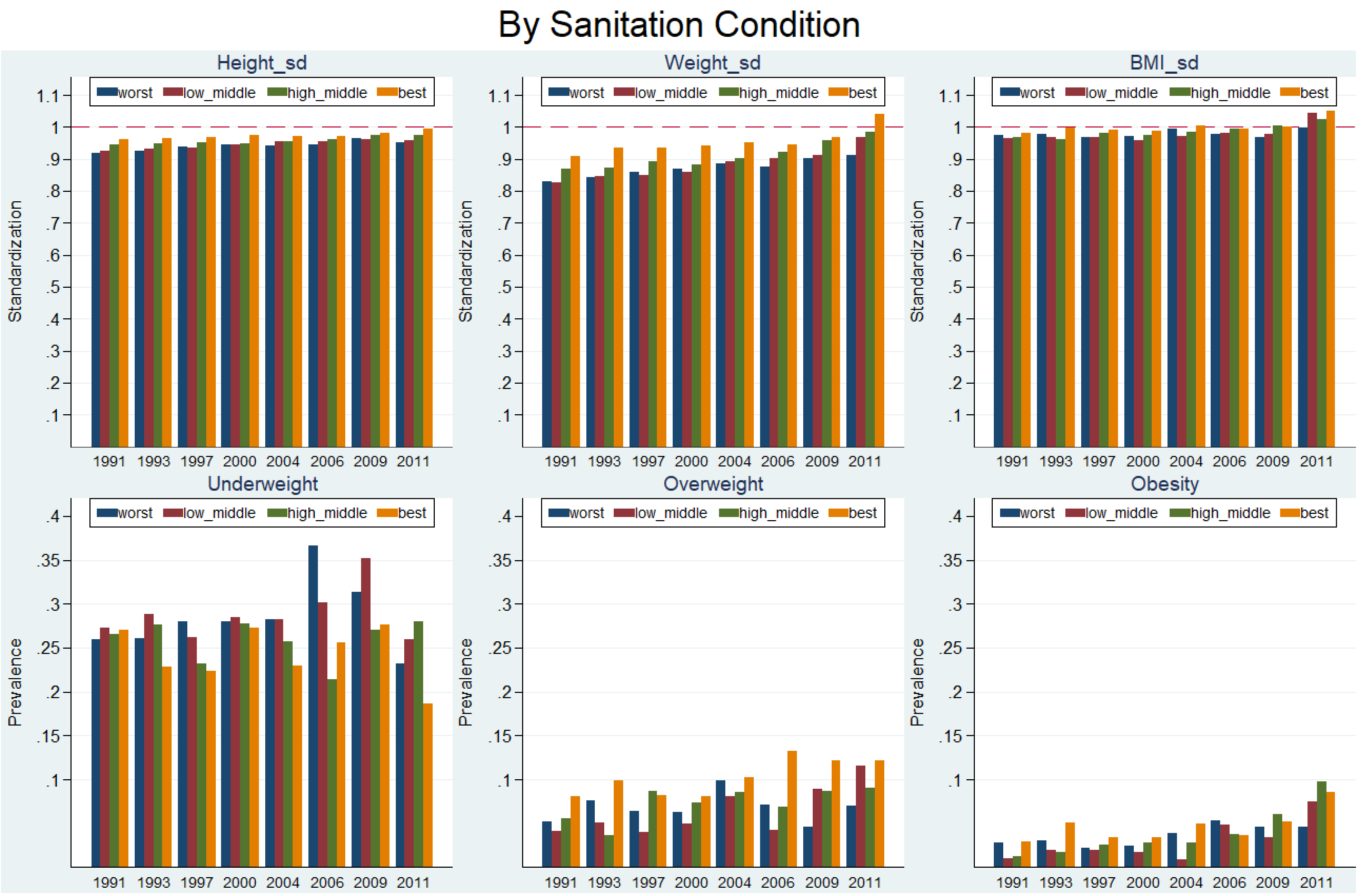

Note: The sanitation condition of household was measured by three variables: drinking water (in-house or in-yard tap water), toilet (in-house flush or in-house toilet) and basic sanitation (no near house excreta removal). Worst, low_middle, high_middle and best refer to families which meet 0 to 3 sanitation conditions.

Figure S4. Secular trend of children's growth indicator and weight status by sanitation condition.

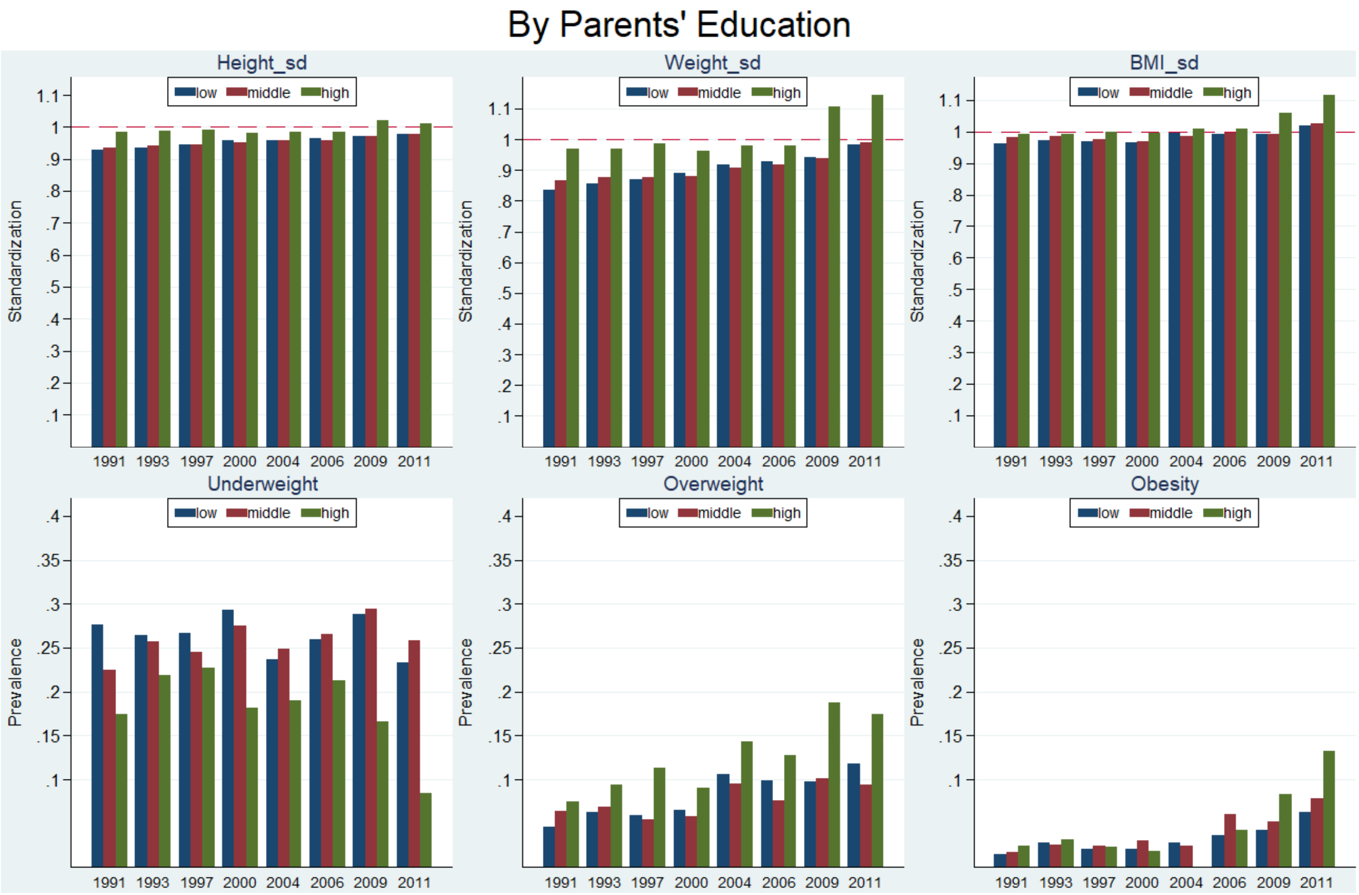

Note: Low, middle and high refer to low education family (none of parents have received more than 6 years formal education), middle education family (at least one of parents have received 6-12 years of formal education but none of them have received more than 12 years of education), and high education family (at least one of parents have received more than 12 years of education).

Figure S5. Secular trend of children's growth indicator and weight status by parents' education.

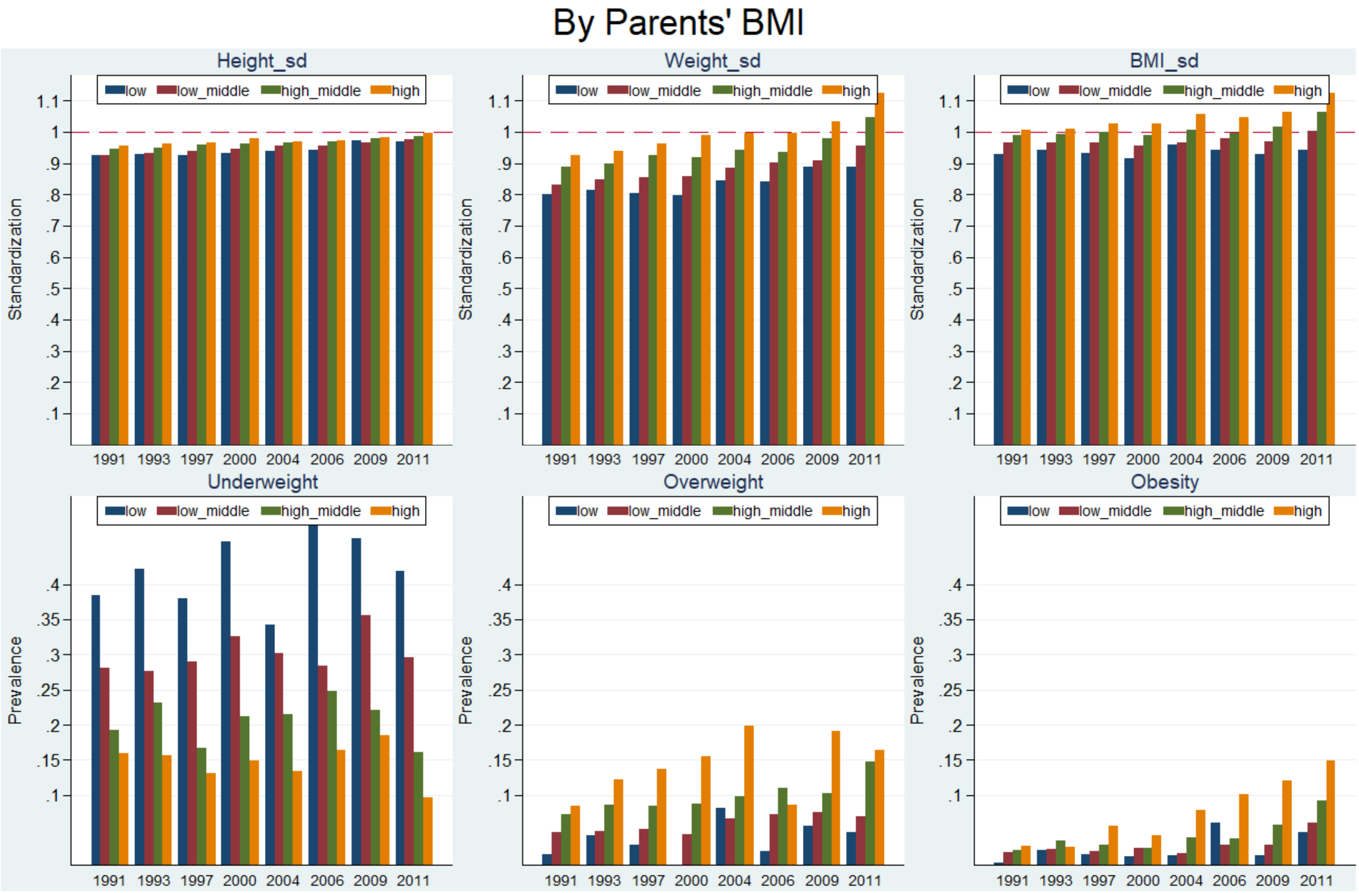

Note: Low, low_middle, high_middle and high refer to 4 family groups: group 1 (none of parents' BMI >= 24 and at least one of them < 18.5), group 2 (one parent's BMI is smaller than 18.5 but another parent's BMI locates between 24 and 28, or both parents' BMI locates between 18.5 and 24), group 3 (both parents' BMI are greater than or equal to 18.5 but smaller than 28, and at least one of them is greater than or equal to 24 but smaller than 28), and group 4 (at least one of parents' BMI >= 28).

Figure S6. Secular trend of children's growth indicator and weight status by parents' BMI categories.
